# Major Discordance in Hemoglobin A1c Measurement in Sickle Cell Trait: Effect of Analytical Methods

**DOI:** 10.7759/cureus.105572

**Published:** 2026-03-20

**Authors:** Faralahy H Rakotonjafiniarivo, Jocia Fenomanana, Soja M Rakotomalala, Aimée O Rakoto Alson, Miora K Ranaivosoa

**Affiliations:** 1 Medical Biochemistry, University of Antananarivo, Antananarivo, MDG; 2 Medical Biology, University of Fianarantsoa, Fianarantsoa, MDG; 3 Medical Biology, University of Antananarivo, Antananarivo, MDG; 4 Hematology, University of Antananarivo, Antananarivo, MDG

**Keywords:** fluorescence immunoassay, fructosamine, hemoglobin a1c (hba1c), high-performance liquid chromatography (hplc), sickle cell trait

## Abstract

Hemoglobin A1c (HbA1c) is widely used for diagnosing and monitoring diabetes mellitus and reflects long-term glycemic control. However, hemoglobin variants may interfere with certain assay methods, leading to misleading results. We report the case of a 54-year-old woman with sickle cell trait and long-standing diabetes who presented with markedly discordant HbA1c values obtained using two different methods. High-performance liquid chromatography (HPLC) showed a significantly elevated HbA1c level, whereas a fluorescence immunoassay yielded a substantially lower value, despite persistently elevated fasting plasma glucose and normal hematologic parameters. Hemoglobin electrophoresis confirmed sickle cell trait. Fructosamine levels were consistent with recent hyperglycemia. This case highlights the potential for hemoglobin variants to interfere with specific HbA1c assay techniques, resulting in inaccurate assessment of glycemic control. Clinicians should interpret HbA1c results cautiously in patients with known or suspected hemoglobin variants and consider alternative markers, such as fructosamine, to guide diabetes management.

## Introduction

Glycated hemoglobin (HbA1c) is a central biomarker in the diagnosis and long-term monitoring of diabetes, as it reflects cumulative glycemic exposure over the preceding two to three months [1]. The clinical reliability of this parameter relies on two essential conditions: a normal red blood cell lifespan and the absence of analytical interference during measurement [2]. However, certain hematological conditions, particularly hemoglobin variants, may affect the accuracy and interpretation of HbA1c results [3]. The global prevalence of sickle cell trait (HbAS), estimated at 2,428 per 100,000 individuals, varies widely across regions, reaching approximately 17,690/100,000 in Africa and only about 212/100,000 in Europe [4]. Different analytical techniques, including ion-exchange high-performance liquid chromatography (HPLC) and immunological methods, exhibit variable susceptibility to interference related to hemoglobin variants [5]. These methodological differences may lead to significant discrepancies in HbA1c values, with potential implications for the assessment of glycemic control and therapeutic management.

We report the case of a patient with sickle cell trait (HbAS) who presented with a marked discrepancy in HbA1c values depending on the assay method, with divergent results between immunofluorescence and ion-exchange HPLC, in the absence of anemia or reticulocytosis. The aim of this study is to illustrate how sickle cell trait can influence HbA1c results and to raise clinicians’ awareness of the need to use alternative glycemic markers when appropriate.

## Case presentation

This case report was conducted in accordance with the ethical principles of the Declaration of Helsinki. Ethical approval was obtained from the local ethics committee, and written informed consent was obtained from the patient for publication of this case report and any accompanying anonymized clinical data.

The patient was a 54-year-old woman with sickle cell trait, confirmed at age 28, who had been followed for 26 years for type 2 diabetes mellitus treated with glimepiride. She reported progressive fatigue over the past month and intermittent bilateral knee pain without morning stiffness, swelling, or inflammatory signs. She denied fever, weight loss, or dyspnea. There was no history of blood transfusion or diabetic complications such as retinopathy, nephropathy, or neuropathy. Further questioning identified opportunities to optimize therapeutic adherence. The patient reported occasional difficulties in taking her medication regularly, maintaining routine follow-up visits, and performing consistent self-monitoring of blood glucose. Vital signs were stable: blood pressure 130/80 mmHg, heart rate 78 bpm, and temperature 36.7°C. Her body mass index was 22 kg/m². Joint examination revealed no swelling, redness, or limitation of motion.

Laboratory evaluation revealed a fasting plasma glucose of 8.97 mmol/L (Table 1). HbA1c measured by HPLC was 10.1%, which was notably discordant with the fasting glucose value. This prompted a repeat HbA1c measurement using a fluorescence immunoassay, which showed a substantially lower value of 7.6%, highlighting a significant difference between the two analytical methods.

**Table 1:** Key laboratory findings Bold values indicate discordant HbA1c results between HPLC and FIA. LDH: lactate dehydrogenase; GGT: gamma-glutamyl transferase; ALP: alkaline phosphatase; CRP: C-reactive protein; HPLC: high-performance liquid chromatography; FIA: fluorescence immunoassay

Parameters	Patient value	Normal range	Unit	Interpretation
Fasting blood glucose	8.97	4.20-6.40	mmol/L	Elevated
HbA1c by HPLC	10.1	<6.5	%	Severely elevated
HbA1c by FIA	7.6	<6.5	%	Elevated
Fructosamine	315	200-286	µmol/L	Elevated
Hemoglobin	131	120-180	g/L	Normal
White blood cells	3.9	4-10	Giga/L	Low-normal
Platelets	167	150-450	Giga/L	Normal
Reticulocytes	63	25-100	Giga/L	Normal
LDH	592	<450	UI/L	Elevated
Total bilirubin	32.2	<21	µmol/L	Slightly elevated
Conjugated bilirubin	2.0	<5	µmol/L	Normal
GGT	18	9-39	UI/L	Normal
Albumin	47	35-53	g/L	Normal
Serum iron	13.2	10-28	µmol/L	Normal
ALP	148	80-360	UI/L	Normal
CRP	1.9	<6	mg/L	Normal
Urea	3.84	2.5-7.5	mmol/L	Normal
Creatinine	72	44-105	µmol/L	Normal
Sodium	149	135-145	mmol/L	High
Potassium	4.9	3.5-5.0	mmol/L	Normal
Chloride	107	98-107	mmol/L	Normal

**Figure 1:**
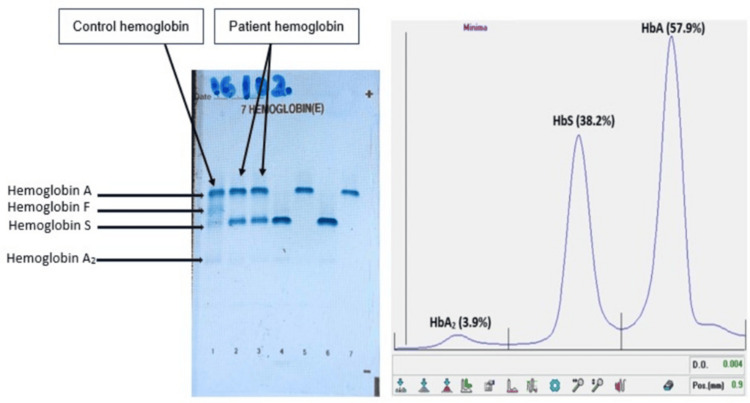
Alkaline pH agarose gel hemoglobin electrophoresis Electrophoresis demonstrating HbA and HbS bands, confirming sickle cell trait (HbAS). The presence of HbS likely contributed to the discordant HbA1c results between analytical methods.

Further investigation, including hemoglobin electrophoresis (Figure 1), confirmed the presence of sickle cell trait (HbAS).

## Discussion

HbA1c and sickle cell trait

In this case, sickle cell trait was confirmed by hemoglobin electrophoresis, which demonstrated a predominance of HbA and the presence of HbS (38.2%). The absence of major clinical manifestations, anemia, or reticulocytosis is explained by the fact that individuals with sickle cell trait generally have hematological parameters (hemoglobin level, hematocrit, and red blood cell indices) that remain within or close to normal ranges, reflecting the typically benign nature of this condition. Sickle cell trait (HbAS), resulting from the inheritance of one normal HbA allele and one HbS allele, is generally considered a benign condition, without vaso-occlusive crises or chronic anemia as observed in homozygous sickle cell disease, and with an overall normal erythrocyte lifespan [6]. In most HbAS individuals, red blood cell survival is comparable to that of HbAA individuals, and HbA remains the predominant hemoglobin fraction [6]. In this context, HbA1c would theoretically be expected to accurately reflect mean glycemia. In the present case, the elevation of lactate dehydrogenase (LDH) and the slight increase in total bilirubin, which are classical biomarkers of hemolysis, reflect red blood cell destruction and may have a direct or indirect impact on certain laboratory tests [7]. However, increasing evidence indicates that the presence of HbS may interfere with certain HbA1c assay methods, independently of anemia or reticulocytosis [8]. The discrepancy observed between the HbA1c values obtained using the two techniques in this case, therefore, appears highly likely to be of analytical origin. Thus, hemoglobin electrophoresis was performed in response to the discordant results and to exclude the presence of an associated hemoglobinopathy. The analysis confirmed sickle cell trait, with no additional hemoglobin variants detected.

Analytical interference of HbS in HbA1c measurement

In the present case, a marked discrepancy was observed between HbA1c values measured by HPLC and by immunofluorescence, despite an elevated mean plasma glucose level (8.97 mmol/L). HbA1c measured by HPLC reached 10.1%, whereas the value obtained by immunofluorescence was significantly lower at 7.6%. This discrepancy is consistent with previously described analytical interferences in individuals with sickle cell trait. HbA1c assay methods rely on distinct analytical principles and show variable susceptibility to interference from hemoglobin variants [9]. Interference from HbS in HbA1c measurement varies by analytical method. Some HPLC-based methods may show a marked negative bias (about −38.9% at 6.5% HbA1c and −41.5% at 9.0%), whereas immunoassay methods generally show lower biases (approximately −1.9% at 6.5% and up to −6.0% at 9.0%) [5]. Ion-exchange HPLC separates hemoglobin fractions according to their electrical charge, allowing differentiation of HbA1c from HbA0 and subsequent quantification of HbA1c based on chromatographic peak integration, standardized according to the Diabetes Control and Complications Trial (DCCT) and the International Federation of Clinical Chemistry and Laboratory Medicine (IFCC) recommendations [10]. The presence of HbS or its glycated forms may alter hemoglobin fraction separation in ion-exchange HPLC. If these variants are not adequately resolved from the HbA1c or HbA0 fractions, peak integration may become inaccurate, leading to falsely elevated or falsely decreased HbA1c results [11]. Conversely, immunofluorescence methods rely on the recognition of the glycated N-terminal epitope of the β-chain of HbA by specific antibodies. The substitution of glutamic acid by valine at position β6 in HbS may alter the three-dimensional structure of the β-chain and potentially affect antibody affinity for the targeted epitope [12]. In HbAS individuals, the reduced proportion of normal HbA, combined with possible altered immunological recognition, may result in artificially low or non-interpretable HbA1c values. In this case, given the major discrepancy between the two HbA1c methods, fructosamine was measured to ensure reliable monitoring of glycemic control. The fructosamine level of 315 µmol/L was consistent with an estimated mean glucose of approximately 8.97 mmol/L, suggesting recent hyperglycemia over the preceding two to three weeks, while acknowledging the inherent limitations of conversion formulas [13]. The use of alternative glycemic markers such as fructosamine, which reflects mean glycemia over the previous 2-3 weeks and is not affected by hemoglobin variants, should therefore be considered for more accurate diabetes monitoring in this patient [14,15].

Clinical and biological implications

Major discrepancies in HbA1c measurement can have significant clinical consequences, including misclassification of glycemic control and inappropriate therapeutic decisions. In patients with hemoglobin variants, HbA1c interpretation should consider the analytical method used and its known limitations. In cases of major inter-method discordance, HbA1c results may be unreliable, and neither value should be considered definitive [11,16]. Alternative markers, such as fructosamine, should be used to guide glycemic management. Additionally, suboptimal medication adherence is a common contributor to poor glycemic control in patients with type 2 diabetes [17], emphasizing the importance of strategies to assess and improve adherence in clinical practice.

## Conclusions

This case highlights that hemoglobin variants may cause significant analytical interference in HbA1c measurements, leading to clinically discordant results. It underscores the importance of selecting an appropriate HbA1c measurement method, as different analytical techniques may be variably affected by hemoglobin variants. When discordant results occur, alternative markers of glycemic control should be considered, and close collaboration between clinicians and laboratory specialists is essential for accurate interpretation and appropriate patient management.
